# P-17. Outcomes of Patients with Suspected Central Venous Catheter-Related Bloodstream Infections Receiving Antimicrobial Lock Therapy for Catheter Salvage

**DOI:** 10.1093/ofid/ofaf695.248

**Published:** 2026-01-11

**Authors:** Emerald O’Rourke, Joseph Corbino, Michelle Lee, Martina Boda, Leonard Mermel, Francine Touzard Romo

**Affiliations:** Brown University Health, Newport, RI; Brown University Health, Newport, RI; Brown University Health Rhode Island Hospital, Providence, Rhode Island; Brown University Health, Newport, RI; Warren Alpert Medical School of Brown University and Rhode Island Hospital, Providence, Rhode Island; Brown University Health, Newport, RI

## Abstract

**Background:**

Antimicrobial lock therapy (ALT) is utilized as an adjunct to systemic antibiotics when catheter salvage is pursued for patients with central venous catheter-related bloodstream infections (CVCRBSIs); however, evidence supporting this practice remains limited. This retrospective case series describes the clinical characteristics and outcomes of ALT for the treatment of suspected CVCRBSIs to achieve central line salvage at a three-hospital health system.

**Table 1.**
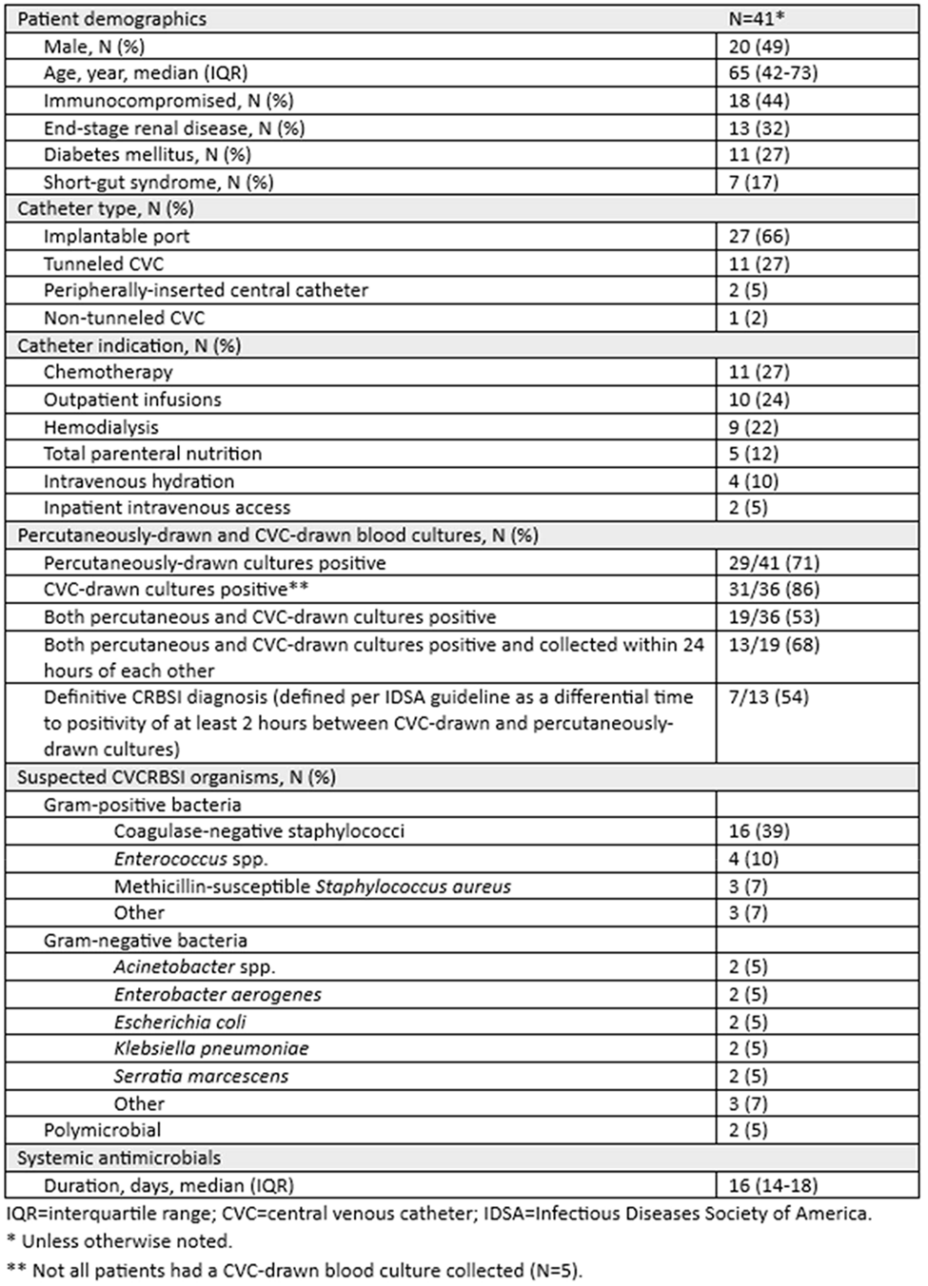
Baseline Characteristics

**Table 2.**
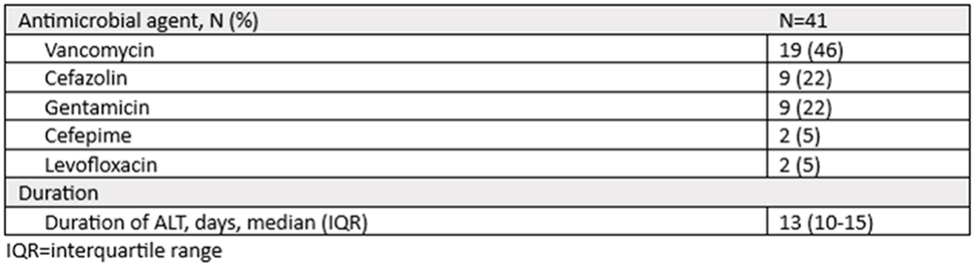
Antimicrobial Lock Therapy

**Methods:**

We identified inpatients ≥18 years of age with an infectious diseases consult and suspected CVCRBSI treated with ≥7 days of ALT with the goal of catheter salvage between April 2016 to November 2024. Patients were included more than once if the subsequent ALT course was caused by a different organism, involving a new CVC, and >90 days from index ALT course completion. Patients receiving ALT as a bridge to CVC removal, prophylaxis, or continuation of outpatient therapy were excluded. The primary outcome was recurrence defined as bacteremia with the index organism within 90 days of ALT completion. Secondary outcomes included bacteremia with a non-index organism, occurrence of CVC removal, and all-cause mortality within 90 days of ALT course completion.

**Table 3.**
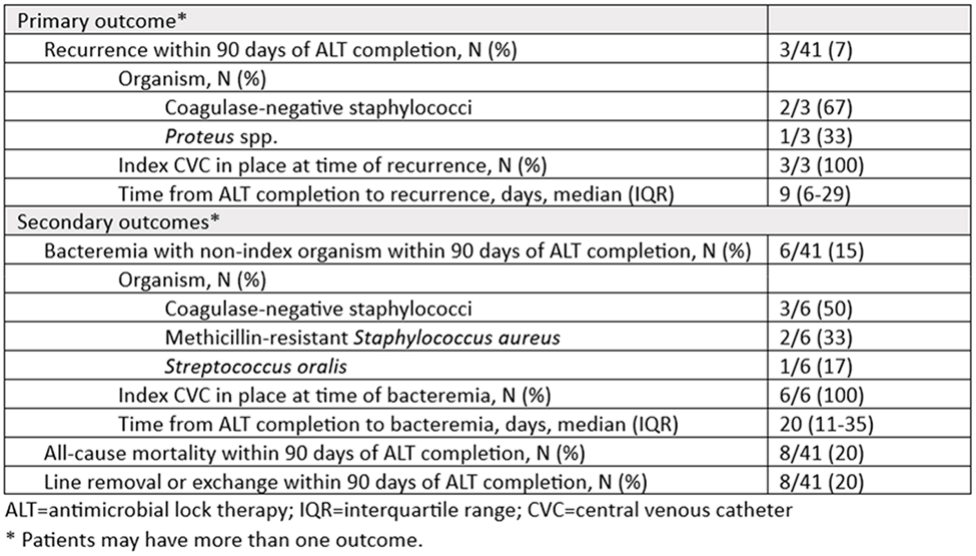
Outcomes

**Results:**

Forty-one ALT courses in 40 unique patients were included (Table 1). Of the 41 suspected CVCRBSIs with CVC-drawn cultures collected, 86% (31/36) had positive CVC-drawn cultures and 53% (19/36) had positive CVC-drawn and percutaneously-drawn cultures. Sixty-three percent (26/41) of suspected CVCRBSIs were caused by gram-positive organisms, 32% (13/41) were caused by gram-negative organisms, and 5% (2/41) were polymicrobial. The median duration of ALT was 13 days (interquartile range (IQR) 10-15 days) (Table 2). Three cases (7%) had a recurrence within 90 days of ALT completion (Table 3). Within 90 days of ALT completion, bacteremia with a non-index organism occurred in 6 cases (15%), line removal or exchange occurred in 8 cases (20%), and all-cause mortality in 8 cases (20%). Catheter salvage at 90 days of ALT completion was successful in 24 cases (59%).

**Conclusion:**

ALT was an effective therapeutic strategy to achieve catheter salvage in over half of our cohort. Larger studies are necessary to optimize the use of ALT and identify factors associated with failure.

**Disclosures:**

Leonard Mermel, DO, Citius Pharma: Advisor/Consultant|CorMedix Pharma: Advisor/Consultant|Destiny Pharma: Board Member|Lightline Medical: Advisor/Consultant|Pristine Access Technology: Advisor/Consultant|Pristine Access Technology: Stocks/Bonds (Private Company)

